# Decisions and Influential Factors Regarding Class-Specific School Closures Against Seasonal Influenza Outbreak

**DOI:** 10.7759/cureus.62394

**Published:** 2024-06-14

**Authors:** Yukiko Masumoto, Hiromi Kawasaki, Miwako Tsunematsu, Ryota Matsuyama, Masayuki Kakehashi

**Affiliations:** 1 School and Public Health Nursing, Graduate School of Biomedical and Health Sciences, Hiroshima University, Hiroshima, JPN; 2 Department of Health Science, Graduate School of Biomedical and Health Sciences, Hiroshima University, Hiroshima, JPN; 3 Department of Health Informatics, Graduate School of Biomedical and Health Sciences, Hiroshima University, Hiroshima, JPN; 4 Department of Veterinary Medicine, School of Veterinary Medicine, Rakuno Gakuen University, Ebetsu, JPN

**Keywords:** elementary school, questionnaire survey, infectious disease control, seasonal influenza, school closure, class-specific school closures

## Abstract

Background

One of the characteristics of school closure in Japan is class-specific school closure, which involves a reactive, short-term closure in the event of an infectious disease outbreak. These closures are implemented at each school in reaction to the annual seasonal influenza outbreaks. Very little research has addressed the formation of class-specific school closures to combat infectious diseases in elementary schools. We carried out a survey on factors involved in the decision to close classes and the determination of the timing and duration of class closures in elementary schools in Japan.

Methods

A mail-based questionnaire survey of elementary schools from four prefectures in western Japan was conducted between August and September 2021. The questions addressed the criteria for school closures (the timing and duration of class closure), various considerations, and confusion regarding class closures, with answers analyzed using descriptive statistical methods.

Results

In total, 714 elementary schools responded to the survey (37.9%). Furthermore, 398 (55.7%) schools established criteria for class closures during seasonal influenza. Class closure was most frequently initiated in schools with criteria when either 20% or 30% of class pupils were absent; the most common duration was three days. The duration of class closures was decided upon depending on the outbreak in some schools (69.8%), depending on the circumstances of the outbreak. Regarding class closure decisions, schools viewed school physicians’ opinions as a priority, followed by school events, adjustments for Saturdays and Sundays, and *Yogo* teachers’ opinions. Schools answering "no criteria for class closure" or "adjustments for Saturdays and Sundays" had difficulty determining class closure duration.

Conclusion

To guarantee the continuation of children’s education and improve the effectiveness of preventive efforts against seasonal influenza, the following were considered important and helpful in class closure decision-making in elementary schools: scientific evidence, the school physician’s opinion, and *Yogo* teachers’ analysis of children's health information.

## Introduction

Among the measures used to prevent infectious diseases in schools is “school closure” [1,2], which is particularly useful in protecting children who are vulnerable to infectious diseases. One of the characteristics of school closure in Japan is class-specific school closure (hereinafter, class closure), which involves a reactive, short-term closure in the event of an infectious disease outbreak. These closures are less burdensome for children and parents [3]. At the same time, they also protect children's health and education and have been implemented during annual seasonal influenza outbreaks [4,5] and periods of pandemic subsidence, such as the 2009 H1N1 [6] pandemic and the COVID-19 pandemic [7]. Many class-based infections occur in elementary schools [8,9]; indeed, one year before COVID-19 became a pandemic, during the 2018/19 seasonal influenza outbreak (November to March), a total of 25,000 class closures were implemented in elementary schools nationwide [10].

Temporary school closures to combat infectious diseases are regulated by the School Health and Safety Law [11], while the school's establishment decides on school closures. In general, the principal of each school decides on the school closure after consulting with the school physician, *Yogo* teachers, and general teachers, as well as obtaining approval from the school's establishment, i.e., the prefectural or city board of education in public elementary schools. In a survey of elementary school principals [12] regarding class closures throughout the seasonal influenza epidemic, school physicians and *Yogo* teachers were the most frequently consulted stakeholders during closures. A *Yogo* teacher is assigned to each school full-time [13] and supports pupils’ growth and development through health education and health services [14,15]. Closing a school is difficult for school administrators, principals, and teachers because it leads to interruption of educational activities and childcare issues [16,17].

The effects of school closures to combat seasonal influenza are controversial [1,2]. Few studies have quantitatively evaluated class closures in response to the seasonal influenza epidemic [2,5], nor has the effect of class closure been clearly demonstrated [5]. As a result, the best strategy for class closure is unclear. Furthermore, few studies have evaluated decision-making regarding class closures in schools, and schools’ experience with class closures is unclear; nevertheless, schools are forced to make decisions under such circumstances. Therefore, the experience of schools regarding class closure during the spread of infectious diseases while facing the dilemma of continuing educational activities may serve as a useful reference for future class closure decision-making.

In the present study, we aim to clarify the situation of class closure during seasonal influenza epidemic periods. We surveyed the criteria for elementary school class closures, the timing and duration of class closures, and the factors considered by the schools when making decisions regarding class closures. This study also assesses the factors influencing the decision on elementary school class closures.

## Materials and methods

Data collection and participants

The survey was conducted between August and September 2021. The inclusion criteria were as follows: all public elementary schools' *Yogo* teachers in the Hyogo, Okayama, Hiroshima, and Yamaguchi prefectures of Western Japan, who agreed to participate. Exclusion criteria were private and special needs education schools. *Yogo* teachers are uniquely licensed educators who support children’s growth and development through health education and health services, such as health observation, first aid, health checkups, health counseling, and hygiene control services in all areas of educational activities in school and are assigned as full-time staff in all elementary, middle, and high schools [14,15]. Principals refer to the professional opinion of *Yogo* teachers as one of the factors to consider for class closure [12]. This study asked *Yogo* teachers to complete a questionnaire to investigate class closures.

A written request and an anonymous questionnaire were mailed to the principals of 1,889 public elementary schools in the four prefectures. The purpose and ethical considerations of the study were explained in the request letter. The letter stated that participation in the survey was voluntary and that no repercussions would result from non-participation. It was also stated that completing the questionnaire would be considered the provision of consent for study participation. After obtaining the consent of the principals, *Yogo* teachers who agreed to participate in the study responded to the survey form and returned it via mail.

The study was approved by the Hiroshima University Epidemiology Research Ethics Committee (E-2532; July 27, 2021).

Survey questions

To determine the characteristics of the survey population, we recorded the number of classes, the number of pupils, and the population of the locality. The *Yogo* teachers were asked about their age, years of experience in the role, and whether they had experienced class closures due to seasonal influenza epidemics. The data did not contain personal information such as names, addresses, or the school where they were employed.

Elementary schools were asked about the factors they consider when deciding the timing and duration of class closures, including criteria for implementing them, various considerations, and opinions of the *Yogo* teachers. The respondents were asked to indicate the timing of the initiation of the closure in terms of the incidence rate per class and the closure duration in terms of the number of days, with or without Saturdays and Sundays. A total of 10 factors were taken into consideration when deciding on class closure, including (1) securing class time (avoiding the loss of class time), (2) school events, (3) adjustments for Saturdays and Sundays, (4) opinion of the school physician, (5) parental requests, (6) opinion of *Yogo* teachers, (7) cancellation of school lunches, (8) stay at home without parents (care for children), (9) increased absenteeism at nearby schools, and (10) class closures at nearby schools (highly considered, somewhat considered, not very considered, or not at all considered).

The opinions of *Yogo* teachers regarding the timing of initiation of class closures were expressed in terms of (1) number of absentees in classes, (2) number of symptomatic cases among those present, (3) status of pupils' use of the health center (*Yogo* teacher’s office room), (4) official documents and guidelines from the Board of Education, (5) recent school epidemics of infectious diseases identified by the national surveillance system “Automatic Information Sharing System for School Absentees” [18], (6) *Yogo* teachers’ knowledge of infection prevention (including environmental hygiene), and (7) knowledge of influenza. We also enquired how frequently the *Yogo* teachers expressed their opinions (often express an opinion, sometimes express an opinion, infrequently express an opinion, or do not express an opinion). Furthermore, we asked questions regarding the difficulty experienced by the schools when it came to the implementation of class closure, including (1) the difficulty in determining the timing of initiation of class closure and (2) the difficulty in determining the duration of class closure (difficult, somewhat difficult, a little difficult, or not difficult).

Analytical strategy

The characteristics of the participants and elementary schools were tabulated and presented as means with distributions. Missing values were excluded from the analysis.

The situation at the implementation of class closure regarding the timing of initiation and duration was summarized and visualized. To calculate the incidence of disease at the beginning of closure and the duration of closure, we categorized the absenteeism rate as 10%, 20%, 30%, 40%, 50%, 60%, 70%, and 80%, and the duration of closure as one, two, three, four, five, and seven days. To better visualize trends in the criteria, if the absence rate and number of days differed from the group class (numerical value), they were included in the closest group. Responses that did not fall into a group, for example, 10-20% absenteeism, were divided into the proposed groups of 10% and 20%. An absentee rate of 10-15% was divided into groups as follows: first, we assumed that the 10-15% rate indicated an equal probability of 1/6 for each 1% increment. Subsequently, we divided the probability into three groups: 10%, 11-14%, and 15%. The probabilities were included in the closest groups, that is, 10% and 11-14% were included in the 10% group, and 15% was included in the 20% group. Thus, 2/3 and 1/3 of the 10-15% responses were added to the 10% and 20% groups, respectively.

We tabulated the difficulties in implementing class closures, including the difficulty in deciding the timing of the initiation and duration of the closure and the opinions expressed by the *Yogo* teachers at the time of class closure.

Multiple logistic regression analysis was conducted to determine the associations between difficulty in deciding on the class closure and the factors involved in deciding on class closure. Difficulty deciding the timing of the initiation and duration of class closure were used as dependent variables. The independent variables included the presence or absence of closure criteria and the presence or absence of considerations (10 items) at the time of class closure. The difficulty in deciding on class closure was categorized as “difficult” (very difficult, somewhat difficult, and a little difficult) or “not difficult.” Each consideration (10 items) for class closure was recorded as having been considered (highly considered or somewhat considered) or not considered (not highly considered or not considered). Statistical significance was set at a two-sided p < 0.05, while IBM SPSS version 27 (IBM Corp., Armonk, NY) was used for statistical analysis.

## Results

Participants’ characteristics

Table 1 summarizes the participants’ characteristics. There were 714 females, with an average age of 42.7 ± 12.5 years and an average experience of 18.7 ± 12.7 years as a *Yogo* teacher. In total, 611 (85.6%) had experienced class closures due to seasonal influenza.

**Table 1:** Characteristics of participants and elementary schools.

	Number	%
Yogo teachers		
Female	714	100.0
Age (years)		
≤29	166	23.2
30–39	127	17.8
40–49	134	18.8
50–59	234	32.8
60–69	40	5.6
Unanswered	13	1.8
Average	42.7	±12.5
Number of years of experience as a Yogo teacher		
≤9	238	33.3
10–19	135	18.9
20–29	123	17.2
30–39	174	24.4
40–49	28	3.9
Unanswered	16	2.2
Average	18.7	±12.7
Experience with seasonal influenza class closures		
Experienced	611	85.6
Inexperienced	84	11.8
Unanswered	19	2.7
Elementary schools		
Population of the city		
<10,000	39	5.5
10,000–49,999	178	24.9
50,000–99,999	65	9.1
≥100,000	355	49.7
Unanswered	77	10.8
Number of classes in the school		
≤5	82	11.5
6–12	307	43.0
13–18	145	20.3
19–24	97	13.6
25–30	50	7.0
≥31 classes	33	4.6
Number of pupils in the school		
<100	207	29.0
100–299	215	30.1
300–499	136	19.0
500–699	86	12.0
700–899	46	6.4
≥900	23	3.2
Unanswered	1	0.1
The existence or non-existence of criteria for class closure in the school		
Criteria present	398	55.7
Criteria absent	303	42.4
Unanswered	13	1.8

Criteria, timing, and duration of class closures

**Figure 1:**
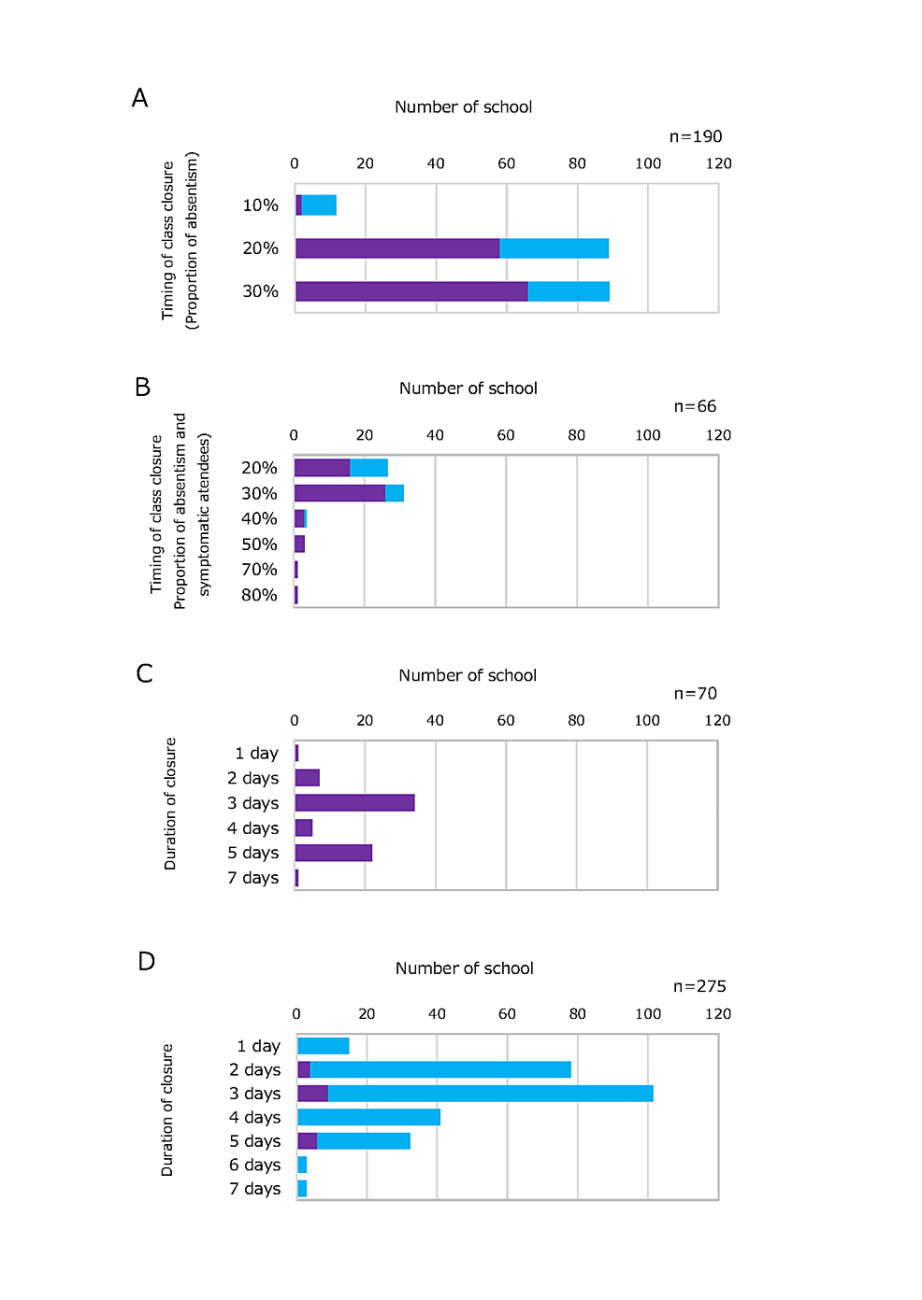
Class closure criteria used by elementary schools: Approximate timing and duration of class closures (with or without Saturdays and Sundays). A: Timing of class closure if solely the proportion of absent pupils in a class was considered. B: Timing of class closure if the proportion of absent pupils and symptomatic attendees were considered. C: Duration of class closure if stipulated days. D: Duration of class closure if case-by-case. Figure A shows the schools where the initiation of class closure was determined solely based on the number of absences. Figure B shows the schools that made decisions regarding class closure based on absent pupils and symptomatic attendees. Figure C illustrates the schools with a fixed number of days of closure. Figure D shows the schools that decided the number of school days closed on a case-by-case basis and the actual number of days of class closure. Purple indicates the real number, and bright blue indicates the calculated overview number.

Criteria for class closures during seasonal influenza were established by 398 (55.7%) schools. The decision regarding the timing of the initiation of class closure was made in 202 schools (50.8%, of which 190 schools presented the percentage) only in terms of the percentage of pupils who were absent from class and in 122 schools (30.8%, of which 66 schools presented the percentage) for a total of the percentage of pupils who were absent from class and those with symptoms. Figure 1 presents criteria for elementary school class closure implementation with estimated timing and duration (with or without Saturdays and Sundays) and uses percentages to depict the timing of class closure. The initiation of class closure in 190 schools where the timing decision was based only on the number of absent pupils in the class was set at an absence rate of 30% of pupils in 89 schools (46.8%) and at an absence rate of 20% of pupils also in 89 schools (46.8%). For 66 schools that made decisions regarding school closure based on the total absence and symptomatic attendance rates, the most common cutoff was 30% of pupils in 33 schools (50.0%), followed by 20% of pupils in 24 schools (35.6%). The duration of each class closure was determined on a case-by-case basis at 275 schools (69.8%), whereas at 70 schools (17.8%), the duration was predetermined using criteria. In both instances, a three-day closure was the most common. Three-day closures were implemented in 101 schools (36.8%) out of 275 schools on a case-by-case basis and in 33 schools (48.6%) out of 70 schools with predetermined criteria.

Factors considered by elementary schools regarding class closure

Table 2 presents the factors elementary schools consider when making decisions regarding class closure. The school physician’s opinion was assigned “very much” importance by 582 schools (83.6%). The factors assigned “very much” and “somewhat” importance included the “opinion of the school physician” in 682 schools (98.0%), the “opinion of *Yogo* teachers” in 611 schools (88.4%), “adjustments for with Saturdays and Sundays” in 535 schools (77.3%), and “school events” in 525 schools (76.1%).

**Table 2:** Factors considered while making decisions regarding class closure.

	Highly considered		Somewhat considered		Not very considered		Not at all considered		Total	
Opinion of the school physician	582	(83.6)	100	(14.4)	12	(1.7)	2	(0.3)	696	(100.0)
Adjustments for Saturdays and Sundays	183	(26.4)	352	(50.9)	120	(17.3)	37	(5.3)	692	(100.0)
School events	156	(22.6)	369	(53.5)	128	(18.6)	37	(5.4)	690	(100.0)
Opinion of Yogo teachers	154	(22.3)	457	(66.1)	73	(10.6)	7	(1.0)	691	(100.0)
Cancellation of school lunches	151	(21.9)	262	(38.0)	200	(29.0)	77	(11.2)	690	(100.0)
Increased absenteeism at nearby schools	147	(21.3)	345	(50.1)	153	(22.2)	44	(6.4)	689	(100.0)
Class closures at nearby schools	145	(21.2)	351	(51.2)	152	(22.2)	37	(5.4)	685	(100.0)
Securing class time	138	(20.0)	358	(51.8)	158	(22.9)	37	(5.4)	691	(100.0)
Stay at home without parents (care for children)	63	(9.2)	280	(40.9)	271	(39.6)	71	(10.4)	685	(100.0)
Parental requests	20	(2.9)	169	(24.5)	345	(50.1)	155	(22.5)	689	(100.0)

Table 3 illustrates the *Yogo* teachers' suggestions regarding implementing class closures. The most frequent suggestions were based on the “number of absences” (94.1%), “number of symptomatic cases among attendees” (84.7%), and “pupils’ use of the *Yogo* teacher’s office” (66.0%).

**Table 3:** Opinion of the Yogo teachers regarding the decision of school closure.

Yogo teachers' opinions	Often express an opinion		Sometimes express an opinion		Infrequently express an opinion		Do not express an opinion		Total	
Number of absentees in classes	571	(94.1)	33	(5.4)	3	(0.5)	0	(0.0)	607	(100.0)
Number of symptomatic cases among those present	515	(84.7)	86	(14.1)	6	(1.0)	1	(0.2)	608	(100.0)
Status of pupils' use of the health center (Yogo teacher’s office room)	400	(66.0)	183	(30.2)	23	(3.8)	0	(0.0)	606	(100.0)
Official documents and guidelines from the Board of Education	244	(40.3)	271	(44.8)	79	(13.1)	11	(1.8)	605	(100.0)
Recent school epidemics of infectious diseases identified by the national surveillance system [18]	242	(40.4)	237	(39.6)	65	(10.9)	55	(9.2)	599	(100.0)
Knowledge of infection prevention (including environmental hygiene)	192	(31.8)	317	(52.5)	91	(15.1)	4	(0.7)	604	(100.0)
Knowledge of influenza	146	(24.2)	309	(51.2)	133	(22.1)	15	(2.5)	603	(100.0)

The relationship between the difficulty of and the factors involved in class closure decisions

Table 4 presents the difficulties faced in implementing the class closure. Difficulty (“very difficult,” “somewhat difficult,” “a little difficult”) was faced by 497 (83.0%) schools regarding the timing of the initiation of closure and by 503 (84.1%) regarding the duration of the closure.

**Table 4:** Determination of class closures.

	Difficult		Somewhat difficult		A little difficult		Not difficult		Total	
Timing of the initiation of closure	125	(20.9)	180	(30.1)	192	(32.1)	102	(17.0)	599	(100.0)
Duration of closure (days)	116	(19.4)	197	(32.9)	190	(31.8)	95	(15.9)	598	(100.0)

Multiple logistic regression analysis was performed to identify the relationships between the difficulty in deciding regarding class closure and the factors involved in deciding on class closure (Table 5). The difficulty in determining the duration of closure significantly differed according to the presence of criteria for the duration of closure (p = 0.025, odds ratio (OR) = 0.55, 95% confidence interval (CI) = 0.33-0.93) and adjustments for Saturdays and Sundays (p = 0.027, OR = 1.85, 95% CI = 1.07-3.19). The relationships between the difficulty in determining the timing of initiation of closure and the factors involved in deciding on class closure were insignificant.

**Table 5:** Association of the difficulty in determining the duration of class closures with the factors involved in deciding on class closures. Multiple logistic regression analysis was conducted by considering the difficulty in deciding on the duration of class closure as the dependent variable (yes: 1, no: 0). CI, confidence interval; OR, odds ratio.

Independent variable		OR	95% Cl	p-value
Criteria for class closure	Yes	0.55	(0.33–0.93)	0.025
Considerations for elementary schools in deciding regarding closure				
Opinion of the school physician	Yes	0.00		0.999
Adjustments for Saturdays and Sundays	Yes	1.85	(1.07–3.19)	0.027
School events	Yes	1.33	(0.73–2.43)	0.350
Opinion of Yogo teacher	Yes	1.71	(0.90–3.25)	0.102
Cancellation of school lunches	Yes	1.27	(0.74–2.17)	0.379
Increased absenteeism at nearby schools	Yes	0.69	(0.27–1.77)	0.440
Class closures at nearby schools	Yes	1.90	(0.75–4.84)	0.178
Securing class time	Yes	1.15	(0.64–2.04)	0.640
Stay at home without parents (care for children)	Yes	1.51	(0.86–2.66)	0.155
Parental opinions	Yes	1.70	(0.83–3.48)	0.146

## Discussion

Class closures have long been used to prevent the spread of illness during seasonal influenza outbreaks in Japan. In this study, we clarified the situation surrounding class closures and assessed the factors involved in class closure decisions during seasonal influenza epidemic periods in elementary schools.

In this study, the timing of class closure was most commonly implemented when the absentee rate was 20% or 30%. The closure duration was most commonly three days but was decided upon depending on the outbreak in some schools. In 2012, attendance suspension due to seasonal influenza was extended from two days to at least five days and two days after fever resolution [19]. Before this extension of duration, class closures were reported to be effective when implemented when the absence rate was ≥ 10% [20]; alternatively, class closure durations of four to five days are also effective [21]. At the time of this study, the longer duration of attendance suspension compared with that may have shortened the duration of infection, resulting in a shorter period for which class closure is required. However, few studies have been conducted after the extended period of absence was implemented; therefore, compared with studies from before this implementation, the criterion for class closure in the current study was higher in terms of the rate of absenteeism, i.e., later in terms of the start of class closures and shorter in terms of the duration of class closures. Schools may have considered closing for a minimal period. This result suggests that the decision to close classes for seasonal influenza in schools is based on the continuation of educational activities and the deterrent effect of the infection. The timing and duration of reactive class closures due to seasonal influenza have been implemented in elementary schools during influenza epidemics based on many years of experience and were implemented in consideration of the balance between infection prevention and educational activities; indeed, these measures may serve as a reference for future decisions regarding class closures. Currently, the effects of class closures have not been quantified [5], and further research is needed to examine the effects of class closures due to seasonal influenza.

This study also considered school events when making decisions regarding class closure. School events are important educational activities for pupils' learning and growth [22], fostering desirable human relationships, promoting cooperation, and developing independent and practical attitudes. On the other hand, children’s routines may change during school closures, and healthy behaviors, such as physical activity or good sleeping habits, may be less likely [23]. The impact of infectious diseases on children's and adolescent's mental health, and responding to the mental health needs of children is more important than ever before [24]. School teachers must consider the negative effects of class closures on the education, growth, health, and nurturing of children and the prevention of the spread of infectious diseases. Therefore, schools face the dilemma of deterring infection and continuing educational activities, which may leave school principals and teachers conflicted when deciding on class closures.

Most schools (69.8%) determined the duration of class closures per school and considered class closures according to the corresponding situation. When deciding on class closures, many *Yogo* teachers expressed opinions about the number of absences, number of symptomatic cases among attendees, and pupils’ use of the *Yogo* teacher’s office, indicating that professionally analyzed pupil health information is needed to consider effective class closures. It is difficult to develop criteria for temporary closure that can be implemented for all schools since the type of infectious disease, the outbreak area, and the outbreak and infection situation vary [25]. In this survey, many schools also considered the timing and duration of class closures on a case-by-case basis. Therefore, the decision to close classes during a seasonal flu pandemic, even for a short period or on a per-class basis, was likely to be difficult and prudent for schools.

Many schools (83.8%) placed substantial importance on the opinion of the school physician when deciding on class closures. These findings align with those of a previous survey [12]. School physicians are expected to provide medical opinions, up-to-date knowledge, medical information, and guidance regarding infectious diseases that are relevant to schools [25]. Seasonal influenza often occurs annually and frequently mutates [26], and schools require readily available consultation, guidance, and expert cooperation [27]. To make a more appropriate choice regarding class closure, we believe that principals and teachers need scientific evidence and the opinions of school physicians about the characteristics and prevention of infection.

Saturdays and Sundays were considered in the decision regarding class closure. Class closures, including Saturdays and Sundays, would avoid impacting educational activities, childcare, and social life. Conversely, it is desirable to avoid an excessive extension of the closing period by including Saturdays and Sundays. In a previous study, seasonal influenza's basic reproduction number (R0) decreased from weekdays to Saturdays and Sundays, indicating a deterrent effect on infection [28]. In addition to experience, school principals and *Yogo* teachers will benefit from understanding the scientific basis for preventing the spread of seasonal influenza, such as the impact of Saturdays and Sundays, to help them make class closure decisions.

In some schools (31.1%), the timing of the initiation of class closure was determined based on the number of absences or symptomatic attendances. This suggests that school teachers pay attention to influenza-like illnesses and absences due to influenza. School teachers systematically and routinely observe the health status of students [11,29]. *Yogo* teachers analyze the number of absences and changes in the students' physical condition daily [14,29] and, in addition to guidelines, they are required to consider infection control measures using their expertise [30]. Therefore, in addition to daily observation of the health status of students, specific education on how to deal with students in the event of an infectious disease outbreak is more important in university education for school teacher licensure, including *Yogo* teachers. By improving the health literacy of school teachers regarding infectious diseases, schools will be able to take advantage of classroom closures and protect students' health and education from infectious diseases.

This study encountered several limitations. First, participation in the survey was voluntary, and the respondents' interest in class closures due to infectious diseases may have been biased. Second, the survey was conducted in four prefectures in western Japan, which may have resulted in regional bias. Third, the survey asked about previous seasonal influenza epidemics; therefore, the experience of simultaneous class closures may have affected the responses due to the COVID-19 pandemic and not during a seasonal influenza epidemic. Finally, we only included *Yogo* teachers in the survey and did not include the opinions of school principals and other teachers. Although it is difficult to generalize the results of this study, our findings may help make decisions and recommendations regarding class closures.

## Conclusions

The results of this study showed that almost half of the schools set class closure criteria. The most common cutoff for initiating class closure was an absence of 20% or 30% of the class pupils. The most common duration of class closure was three days, including or not including Saturdays and Sundays. When making decisions regarding class closure, schools gave the opinion of the school physician the highest priority, followed by school events, adjustments for Saturdays and Sundays, and the opinion of the *Yogo* teachers. Elementary schools that reported difficulty in determining the closure duration were those with no closure criteria and attempted coordination with Saturdays and Sundays.

To achieve the difficult goals of guaranteeing the continuation of children’s education and improving the effectiveness of preventive efforts against the spread of seasonal influenza, the following were considered important and helpful in decision-making on class closure in elementary schools: scientific evidence, the opinion of school physicians, and the analysis of children's health information by *Yogo* teachers.

## Figures and Tables

**Figure 2:**
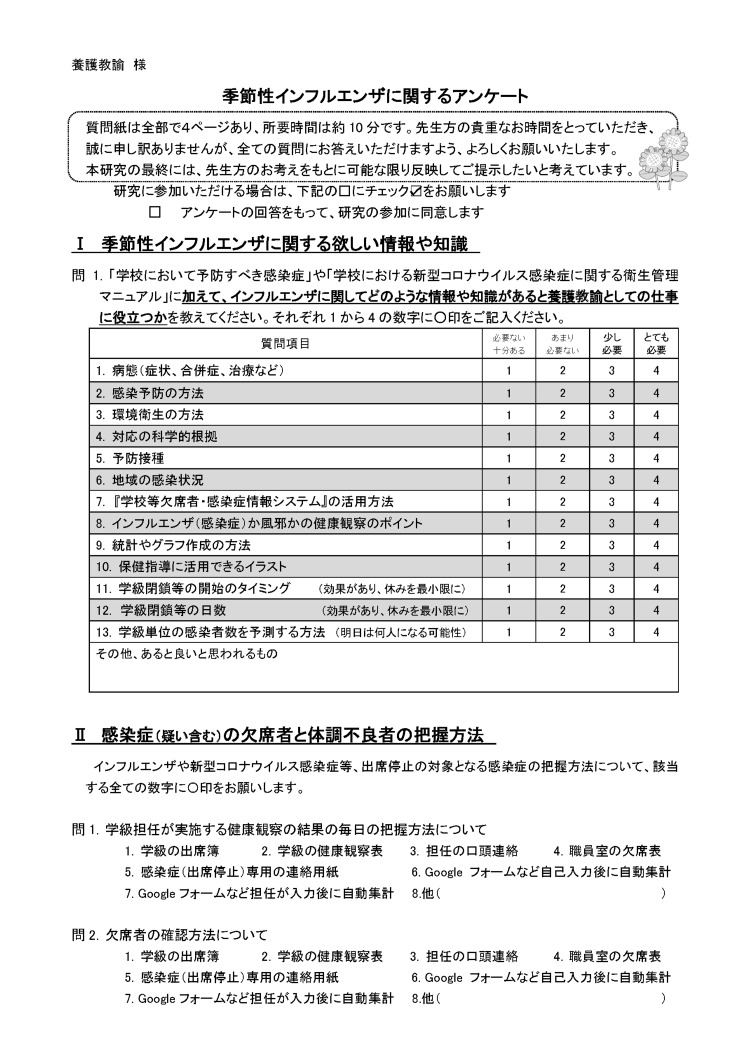
Questionnaire 1.

**Figure 3:**
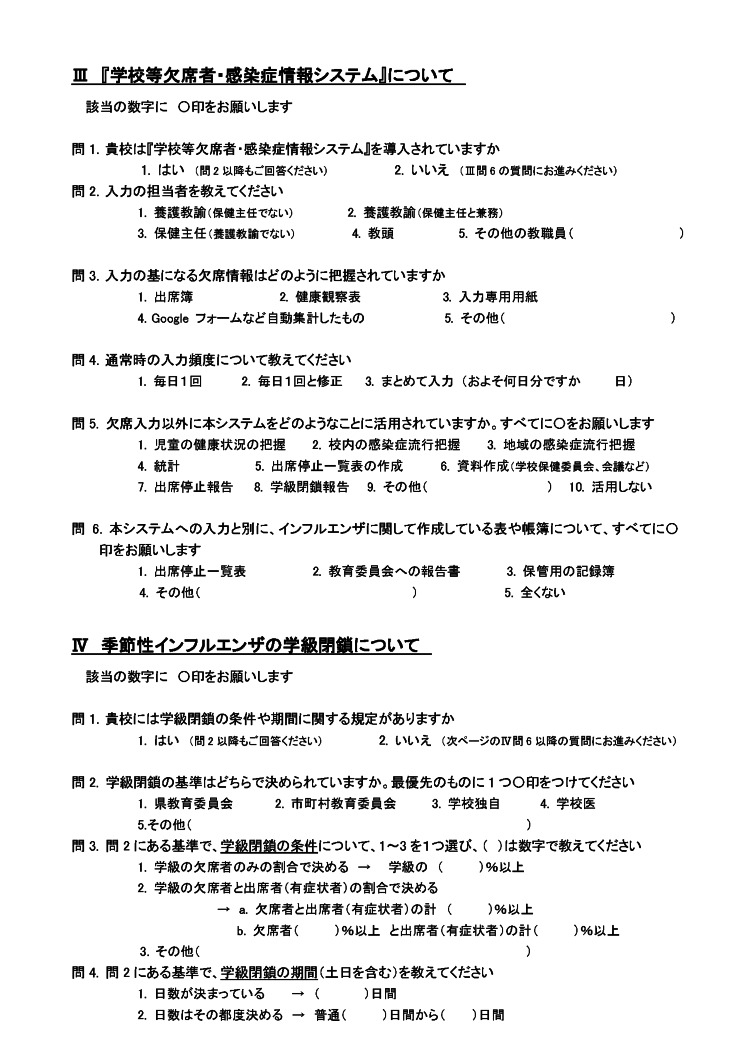
Questionnaire 2.

**Figure 4:**
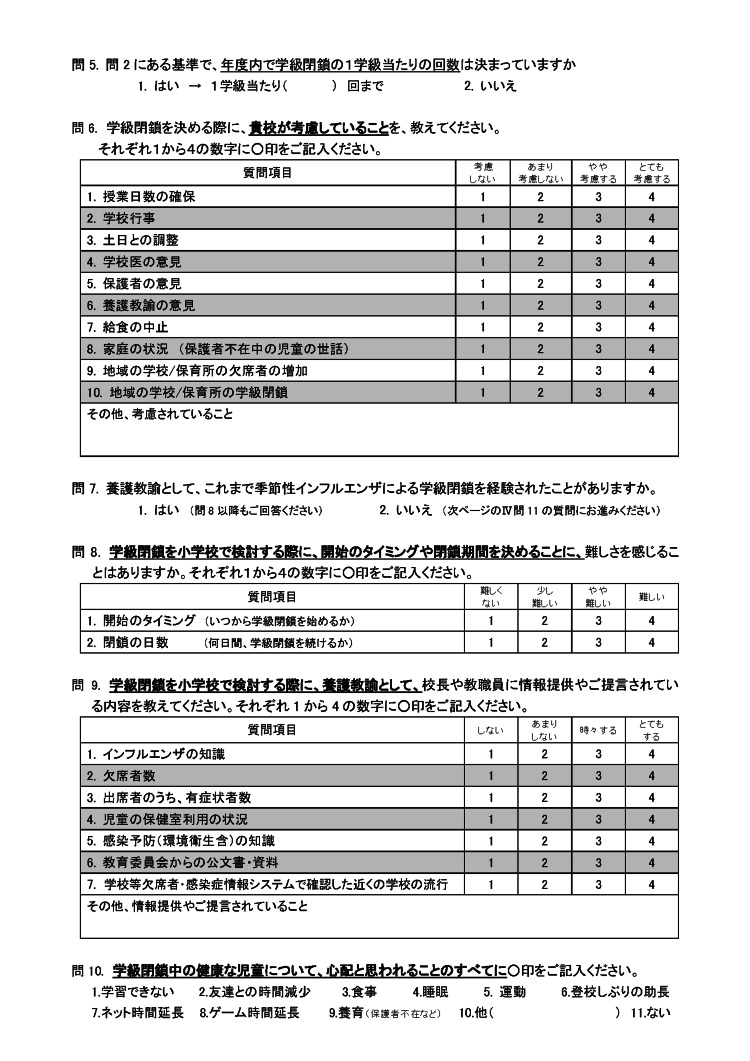
Questionnaire 3.

**Figure 5:**
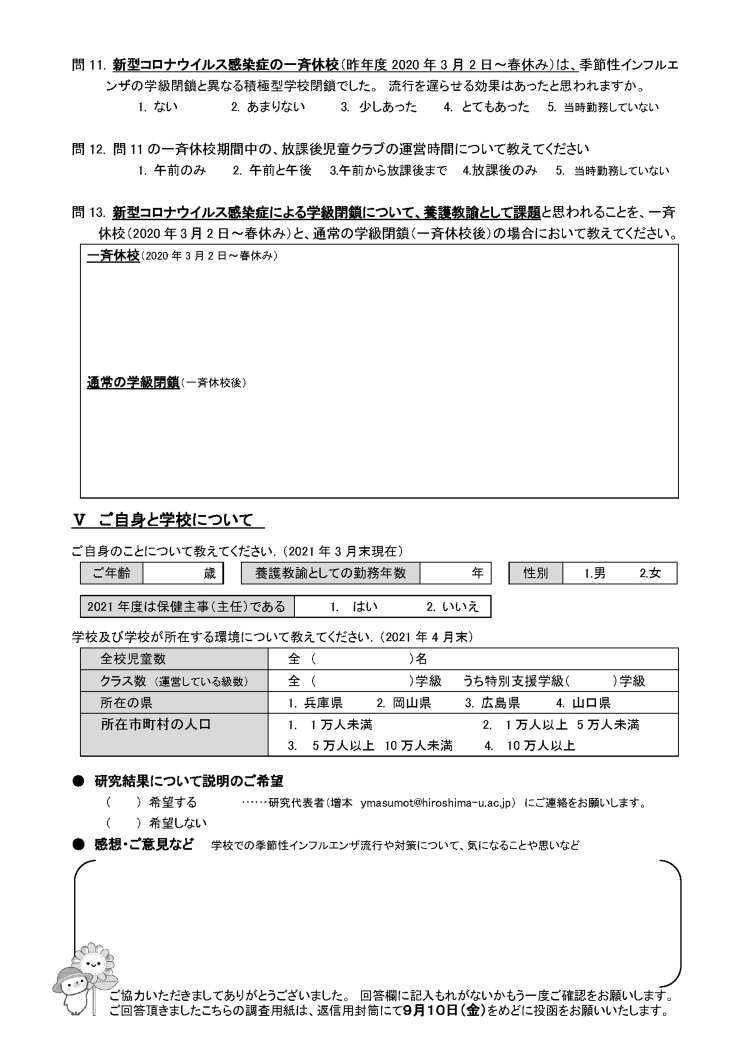
Questionnaire 4.

**Figure 6:**
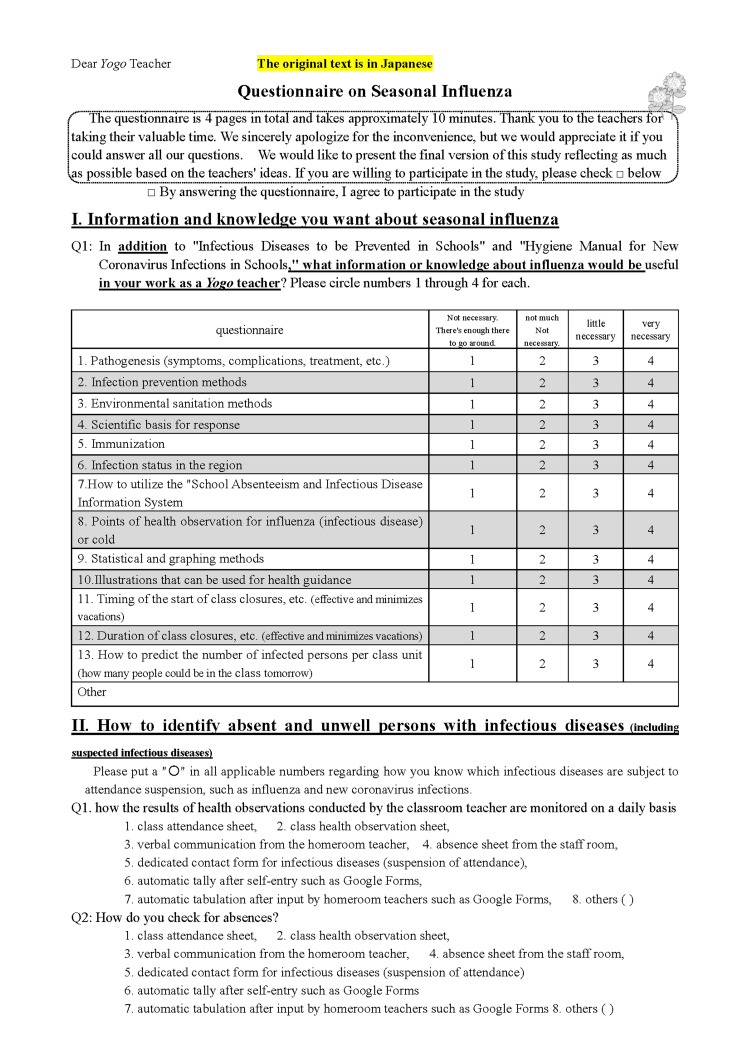
Questionnaire (English translation) 1.

**Figure 7:**
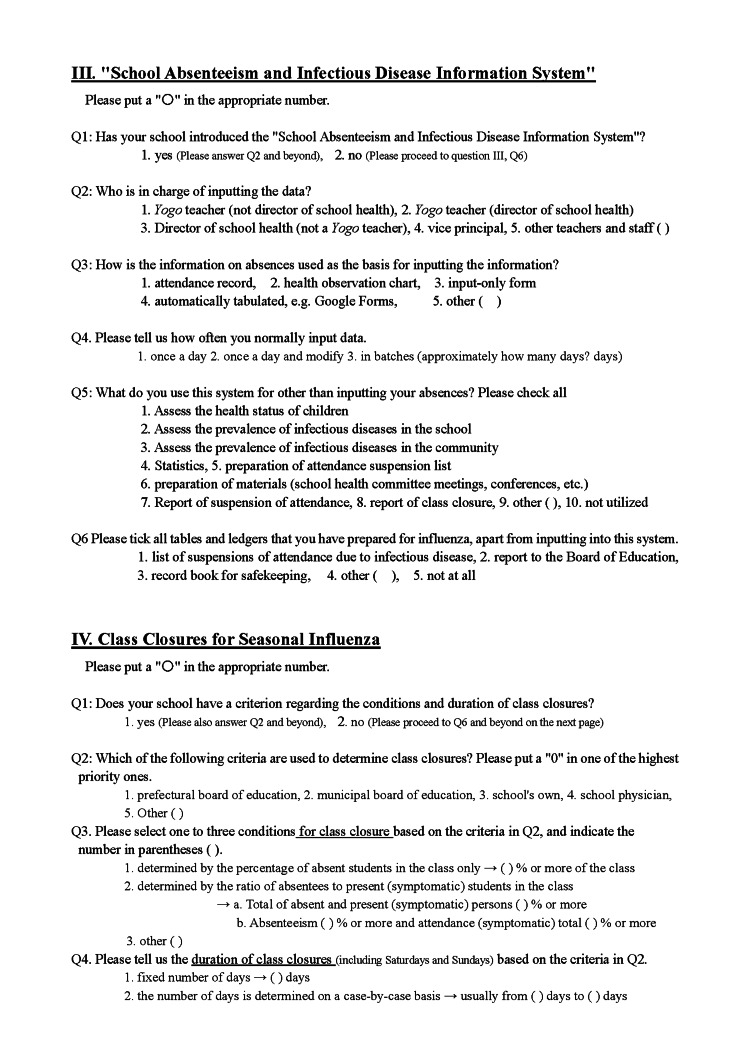
Questionnaire (English translation) 2.

**Figure 8:**
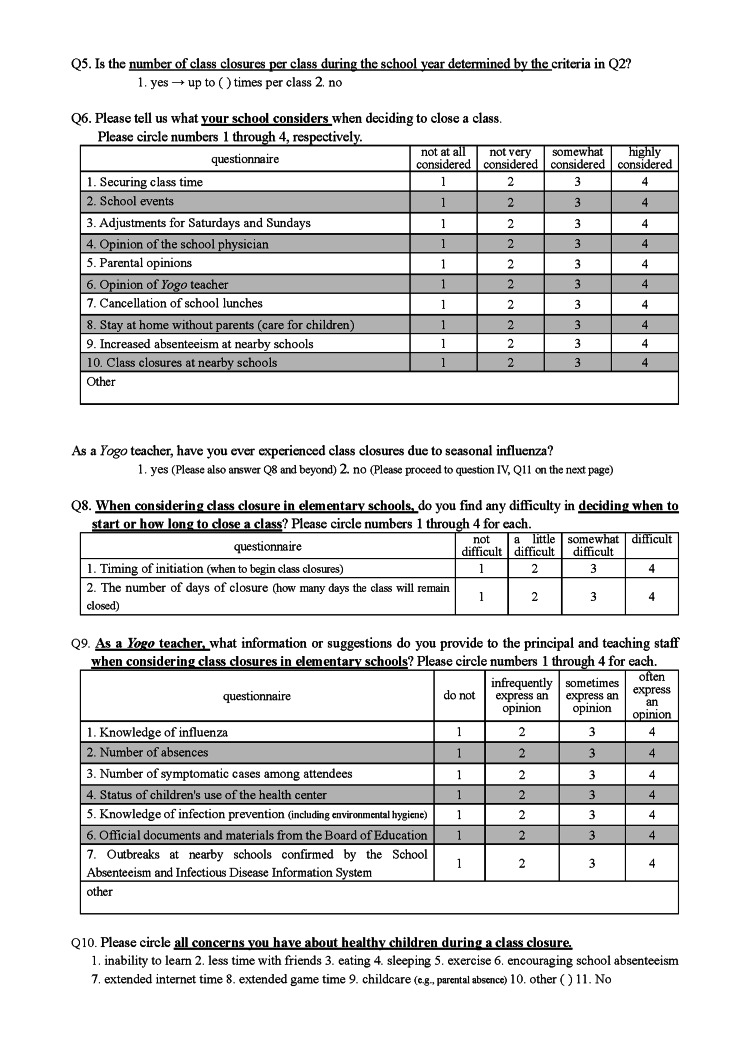
Questionnaire (English translation) 3.

**Figure 9:**
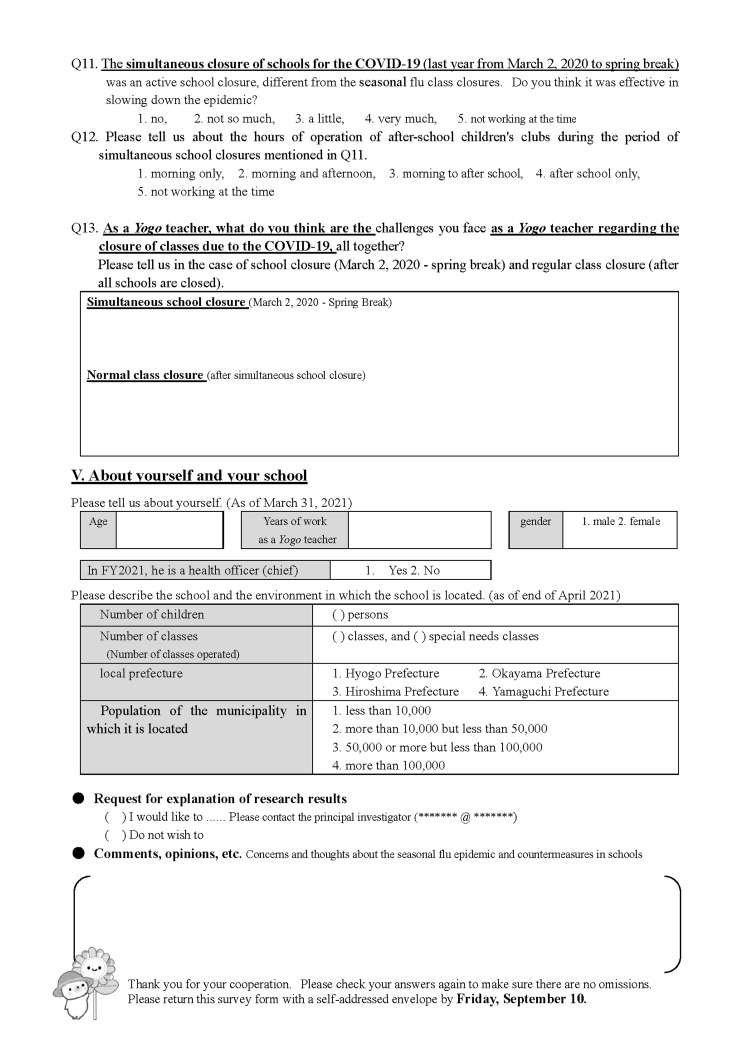
Questionnaire (English translation) 4.

## Appendices

**Table 6:** Criteria for the timing of class closure used by elementary schools. This is a detailed result of the timing criteria presented in Figure 1.

Timing of class closure	Schools	%
a. Percentage of absentees		
Total	190	100.0
10%	2	1.1
10–15%	2	1.1
15%	12	6.3
15–20%	7	3.7
20%	58	30.5
20–30%	37	19.5
25%	2	1.1
30%	66	34.7
33%	2	1.1
34%	1	0.5
35%	1	0.5
b. Percentage of absentees and symptomatic attendees		
Total	66	100.0
15%	2	3.0
20%	16	24.2
25%	2	3.0
30%	26	39.4
40%	3	4.5
50%	3	4.5
70%	1	1.5
80%	1	1.5
15–20%	3	4.5
23%	1	1.5
20–30%	7	10.6
30–40%	1	1.5

**Table 7:** Criteria for the duration of class closure used by elementary schools. This is a detailed result of the duration criteria presented in Figure 1.

Duration of class closure	Schools	%
Number of days		
Total	70	100.0
1	1	1.4
2	7	10.0
3	34	48.6
4	5	7.1
5	22	31.4
7	1	1.4
Approximate number of days for each case		
Total	275	100.0
1	0	0.0
2	4	1.5
3	9	3.3
4	0	0.0
5	6	2.2
6	0	0.0
7	0	0.0
1–2	19	6.9
1–3	15	5.5
1–4	1	0.4
1–5	1	0.4
2–3	100	36.4
2–4	16	5.8
2–5	15	5.5
3–4	22	8.0
3–5	48	17.5
3–7	4	1.5
4–5	7	2.5
5–7	6	2.2
Other	2	0.7

 Questionnaire

Questionnaire (English translation)
